# Transvaginal excision of rectal stromal tumors: case reports and a literature review

**DOI:** 10.1186/s12957-019-1703-8

**Published:** 2019-10-06

**Authors:** Wang Shizhuo, Liuyuan, Ni Sha, Chen Xueting, Wang He, Luan Nannan, Ma Xiaoxin

**Affiliations:** 1 Department of Obstetrics and Gynecology, Shengjing Hospital, China Medical University, 36 San Hao Street, Heping District, Shenyang, 110004 Liaoning China; 2 Department of Gastrointestinal Department, Shengjing Hospital, China Medical University, 36 San Hao Street, Heping District, Shenyang, 110004 Liaoning China

**Keywords:** Rectal GIST, Transvaginal excision, Lower region

## Abstract

**Background:**

Gastrointestinal stromal tumors (GISTs) are the most common mesenchymal tumors of the gastrointestinal tract. Rectal locations are very rare, and minimally invasive surgery is a good choice for the treatment of rectal GISTs.

**Case presentation:**

Two women each had a mass located on the lower vaginal-rectal space as determined by transvaginal ultrasound (TV-US), pelvis MR imaging, and colonoscopy. The patients successfully underwent transvaginal excision. The spindle-shaped cells were found in pathological test. The immunohistochemical analysis showed that CD117 and Dog-1 were stained positively. These results confirmed the masses as GISTs. The postoperative period was uneventful without anal dysfunction. Two patients were received adjuvant treatment with imatinib after surgery.

**Conclusion:**

Transvaginal excision could be a minimally invasive and safe alternative treatment in the management of rectal GISTs in lower locations.

## Background

Gastrointestinal stromal tumors (GISTs) are a common type of mesenchymal tumors. The common sites of origination are the stomach (60–70%) and intestines (20–30%) [1, 2]. Rectal GISTs are rare. Additionally, a rectal GIST is an extremely rare entity in females [3].

Different surgical methods have been widely reported, including transanal excision, laparoscopic surgery, trans-sacral excision, and transanal endoscopic microsurgery (TEM) [4–7]. Sometimes, rectal GISTs, especially those located in the lower rectum, are detected upon expansion of the posterior wall of the vagina during a gynecological examination in females. Therefore, we considered transvaginal resection as an alternative treatment because of the vaginal-rectal anatomical location of the tumors in question. Here, we reported two cases of transvaginal excision at Shengjing Hospital during 201–22018 to highlight the clinical and surgical features of lower rectal GISTs.

## Case presentation

### Case one

**Fig. 1:**
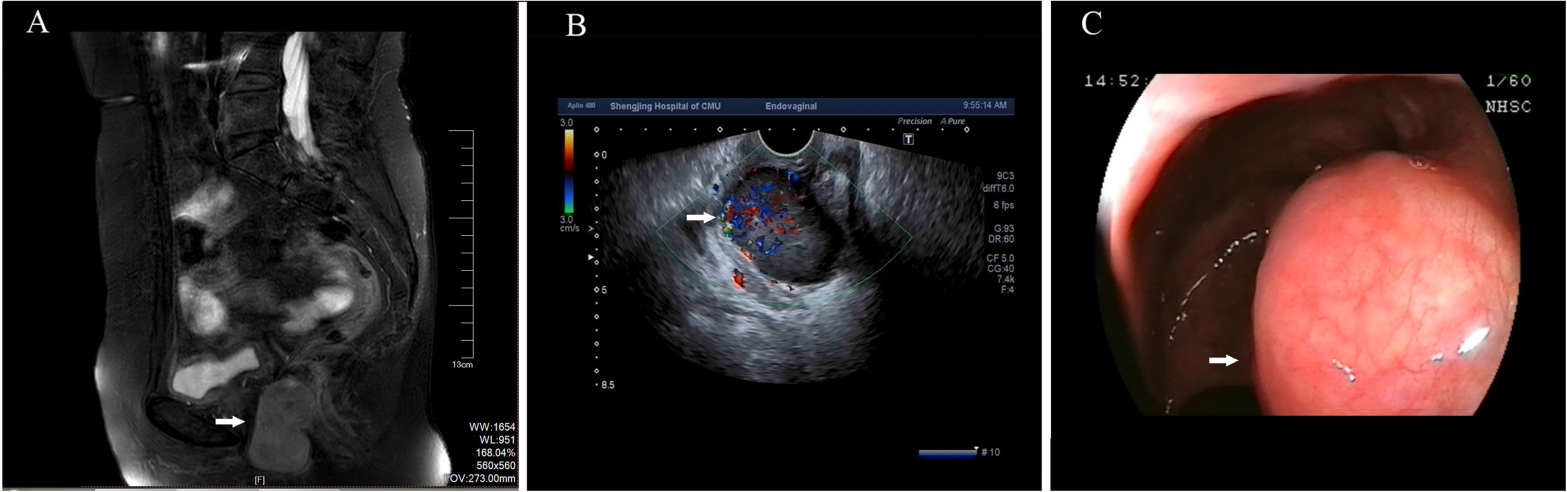
Imaging examinations show the location of the rectal GISTs. **a** The results of pelvic MR imaging reveal that the tumor (arrow) was located in the vaginal-rectal space and protruded from the anterior rectal wall. **b** The results of transvaginal ultrasound show that the tumor (arrow) protruded from the posterior of the lower vaginal wall. **c** Colonoscopy results show that the tumor originated from the rectal dentate line

**Fig. 2:**
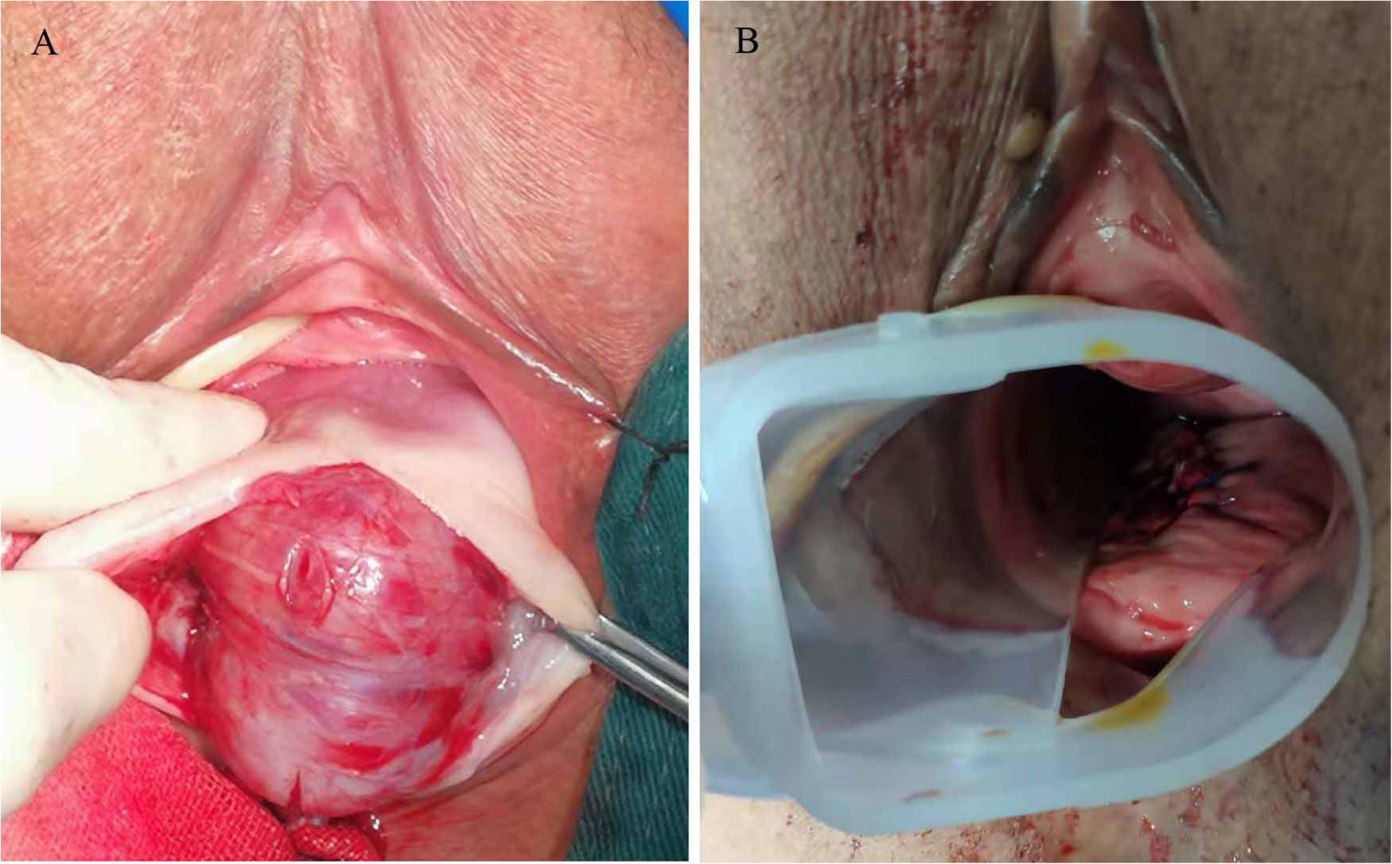
Transvaginal surgery. **a** After resection of the posterior vaginal wall, the tumor (arrows) are completely exposed. **b** After removing the tumor, we stitched the vaginal layers

**Fig. 3:**
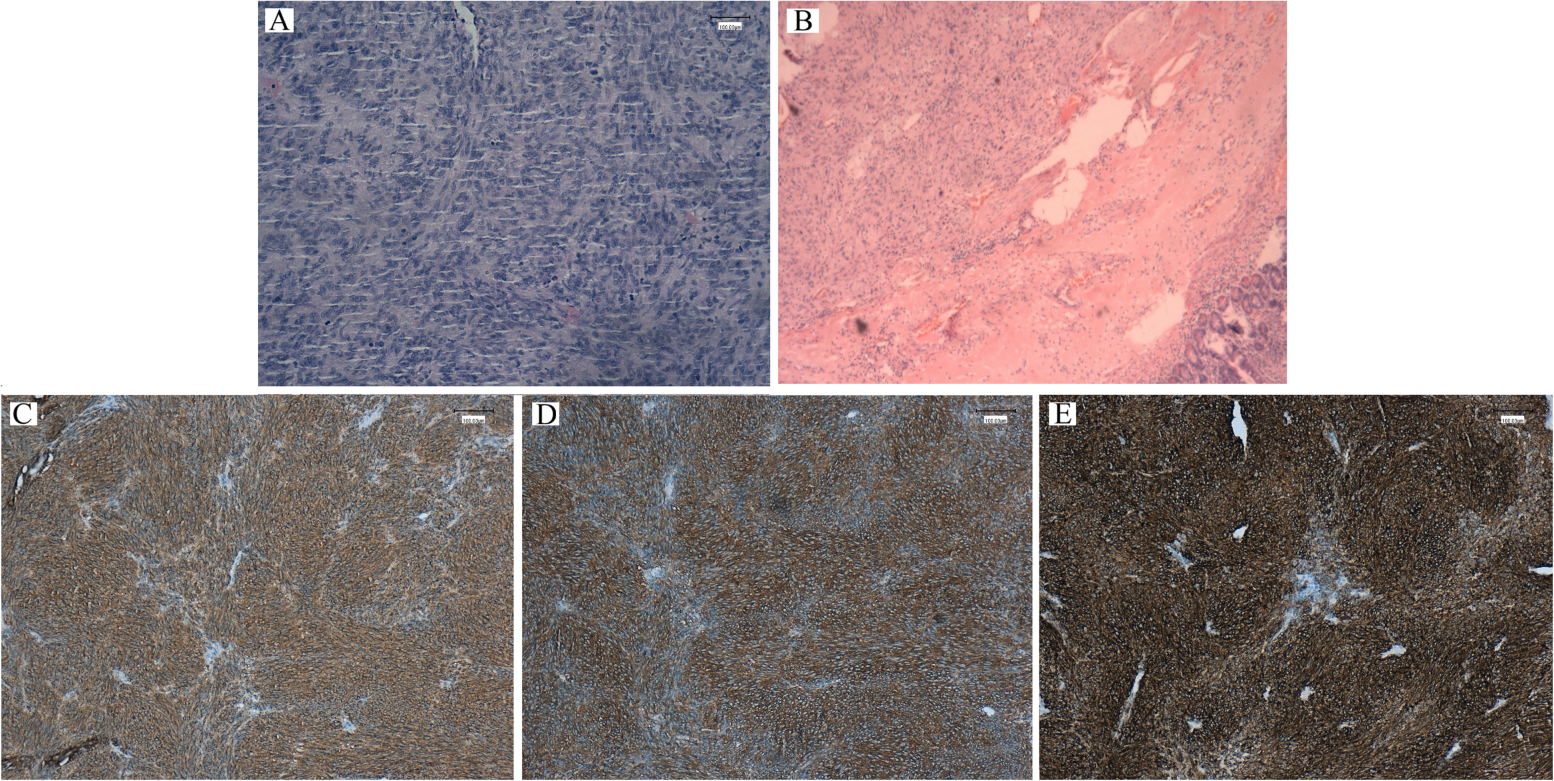
Histopathology of the biopsy specimen. **a** Histopathology showed the cells were shown as spindle-shaped and differentiated moderately. **b** The tumor was resected with a clear margin. Immunohistological results showed that CD117 (**c**), Dog-1 (**d**), and CD34 (**e**) were all positive, indicating a moderate-risk GIST

A 62-year-old woman, G1P1, was referred to a gynecological doctor for a large “vaginal mass”. She did not have abnormal vaginal bleeding but found one vaginal mass by herself 1 month ago. Her age at the beginning of menopause was 52 years old. Her medical and surgical histories were both negative. On gynecological examination, we found that the mass was non-mobile and was 5 × 5 cm^2^ in size, with a location of approximately 3 cm from the vaginal orifice and closely attached to the vaginal wall. On rectal examination, we found that the mass located on the anterior of the rectal wall was approximately 3 cm from the anal verge. The pelvis MR scan and transvaginal ultrasound results showed a tumor, 5 cm in diameter, was mostly located in the space of the rectovaginal septum, with large portion protruding into the vaginal wall but only a small portion protruding into the rectal wall. Its boundary is clear (Fig. 1a, b). Colonoscopy revealed that the root of the tumor was located on the rectal dentate line (Fig. 1c). The origin of the tumor was uncertain. Based on these examinations, the gastrointestinal doctor and us co-evaluated that if we selected a transvaginal resection, we could intactly excised the tumor with less possible complications such as fecal incontinence or anal sphincter dysfunction due to its special location. The patient refused to radical anal resection for its anal complications. Therefore, we chose transvaginal resection as a better alternative. Under general anesthesia, the patient was placed in a lithotomy position. Epinephrine, diluted at 1:40,000, was injected into the vaginal submucosa for resection. We incised the vaginal mucosa and separated the surrounding tissue until we reached the submucosa, keeping the tumor capsule intact. After exposing the tumor, we confirmed that it was located in the rectovaginal septum and partially encapsulated by the rectal muscle (Fig. 2a). We mobilized the tumor from the capsule and resected the intact tumor. The defect of rectal muscle was very small but kept the rectal mucosa intact. We vertically stitched the vaginal layers and horizontally stitched the muscular layer of the rectum (Fig. 2b). The postsurgery biopsy showed spindle-shaped cells were moderate differentiation and regular arrangement with clear margin by pathological examination (Fig. 3a, b). The results of histological examination showed that the tumor was positive for CD117, Dog-1, and CD34 (Fig. 3c, e). These findings suggest a moderate-risk rectal GIST that required follow-up. The patient recovered quickly. She had not suffered any anal dysfunction nor postoperative vaginal-rectal fistula. She refused to undergo enlarged resection but received imatinib treatment after surgery. She remained tumor-free for 2 years after surgery. She was lost for follow-up thereafter.

### Case two

**Fig. 4.**
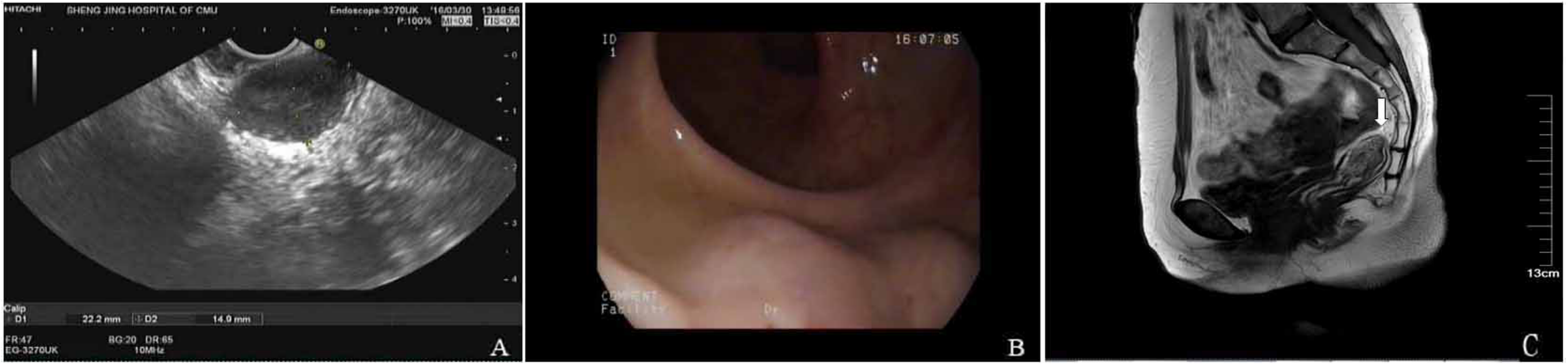
Imaging examination shows the location of the other rectal GIST. **a** Endoscopic ultrasonography shows that the tumor protruded from the anterior rectal wall. **b** The colonoscopy results show that the root of the tumor was located only 3 cm above the dentate line. **c** The results of MR (arrow) showed that the tumor was located in the space of the rectovaginal septum and encapsulated by the rectal muscle, with partially unclear boundary to the vaginal wall

**Fig. 5:**
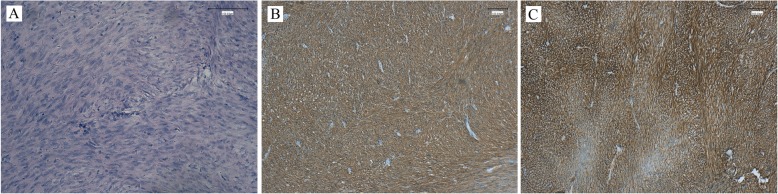
Histological examination shows that cells were spindle-shaped (**a**). The specimen was positive for CD117 (**b**) and Dog-1 (**c**), indicating a low–moderate risk

**Fig. 6:**
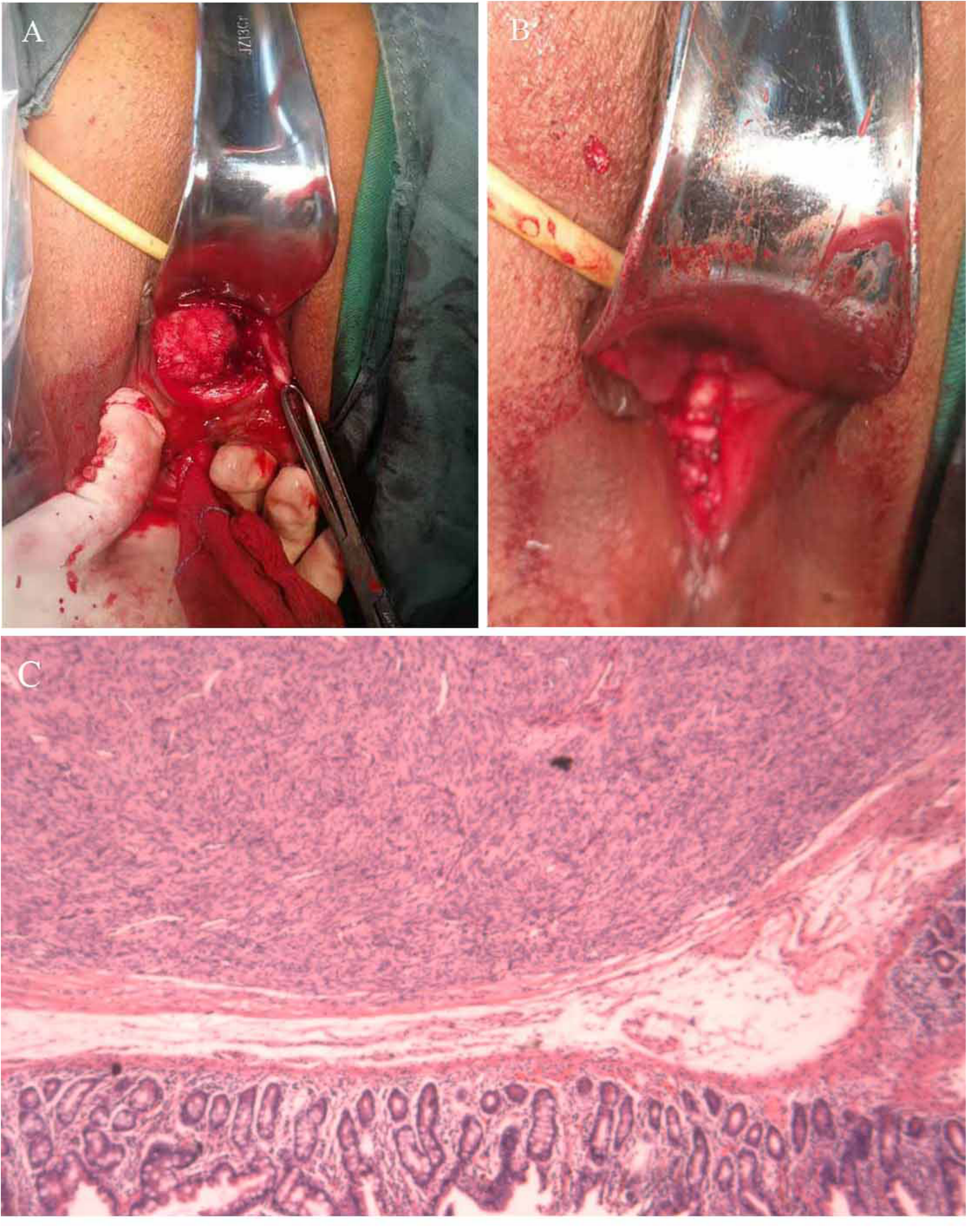
Transvaginal surgery for the second rectal GIST. **a** After resection of the posterior vaginal wall, the tumor was completely exposed. **b** After removing the tumor, we stitched the vaginal layers. The tumor was resected with clear margin (**c**)

We reviewed the relative cases in Shengjing Hospital during 2001–2018. The other GIST patient, a 69-year-old woman with hypertension, was also treated with transvaginal resection. The tumor was 4 cm in diameter, was located in the vaginal-rectal space and protruded from the lower posterior vaginal wall. On gynecological examination, we found that the mass was fixed and closely attached to the vaginal wall. The results of endoscopic ultrasonography showed that only a small portion of the tumor protruded from the anterior rectal wall (Fig. 4a). Colonoscopy revealed that the root of the tumor was located approximately 3 cm above the dentate line (Fig. 4b). The results of pelvic MR imaging showed that the tumor was located in the space of the rectovaginal septum and encapsulated by the rectal muscle, with partially unclear boundary to the vaginal wall (Fig. 4c). The ultrasound biopsy showed that some spindle-shaped cells were irregularly arranged. The cells were stained positive for CD117 and Dog-1 (Fig. 5). The results suggested a low–moderate risk rectal GIST. The patient also refused to radical surgery for her old age and possible anal dysfunction. We suggested the patient consider preoperative imatinib treatment, since it may reduce the tumor volume and improve the chances of radical surgery. However, the patient refused preoperative imatinib treatment. At last, we chose a transvaginal resection as an alternative. The surgical method was similar to that described in the previous case (Fig. 6a). During surgery, we found the tumor was a circumscribed rectal submucosal mass with vaginal submucosa invasion. The tumor was intactly excised with the adherent vaginal wall and the rectal mucosa showed only a 2 × 1.5 cm^2^ defect. We repaired the defect and stitched all the layers (Fig. 6b). The postoperative course was safe and quick. The patient went back home on the fifth day without any anal dysfunction nor vaginal-rectal fistula. The results of postoperative histological examination showed that the tumor was resected with clear margin (Fig. 6c). They were coherent with the results of preoperative biopsy. The patient recovered quickly. She had not suffered any anal dysfunction nor postoperative vaginal-rectal fistula. She also refused radical resection but received imatinib treatment after surgery. She remained tumor-free for 12 months till now.

## Discussion

Intact resection is the best choice for rectal GIST treatment. Radical resection is one of the most important factors for rectal GIST prognosis [8]. However, the surgical procedure for rectal GISTs is difficult and has been debated [9–11]. Most GISTs originate from the muscularis propria and occasionally from the muscularis mucosa [10, 11]. For large rectal GISTs or lower rectal GISTs, radical resection may induce severe anal dysfunction and discomfort. Currently, selecting different surgical procedures for minimally invasive surgery is widely accepted [11, 12]. Increasing research has shown that minimally invasive surgery, such as transanal endoscopic surgery, could reduce the rate of anal dysfunction [13, 14]. The approach of minimally invasive surgery that we choose for rectal GIST patients depends on the tumor pathology, volume, location, and the surgeon’s skills. The most common approach is trans-anal resection. It is suitable for small GISTs located in the distal rectum with limited bowel circumference extension [15, 16]. Trans-sacral resection and TEM are suitable for tumors located on the posterior wall or in the middle or upper rectal areas [7, 17]. When the tumor is located in lower rectum, with a high risk for metastasis or large volume, we can also consider preoperative imatinib treatment for shrinking tumor volume, improving intact resection, good anal functional, and improving disease-free survival [18–20]. Meanwhile, when the tumor is located in the lower rectal part and its large part was encapsulated by the vaginal-rectal septum and protruded into the vaginal wall, we could choose transvaginal resection. There have been few reports discussing the transvaginal resection of rectal GISTs [21, 22]. Hellan et al. first reported that this approach successfully resected larger tumors, sparing the patient from an unnecessary large anal resection [21]. Later, Hara et al. also reported that transvaginal resection of low anterior rectal lesions may provide a minimally invasive alternative to traditional ultra-low anterior resection [22]. In this report, we describe the cases of two successfully resected rectal GISTs located on the anterior rectal wall through a transvaginal procedure. Transvaginal resection is an approach worth considering, as the volume of vagina is large enough to accommodate the resection procedure for GISTs. Both of them received postoperative imatinib treatment to prevent recurrence. These two patients failed to receive preoperative imatinib treatment. One patient did not have preoperative biopsy while the other refused to receive. Cavnar et al. reported that as for moderate–high rectal GIST, preoperative imatinib treatment was associated with higher rates of organ preservation, negative margins, and recurrence-free survival [18–20]. One systematic review reported the significance of preoperative imatinib treatment on rectal GIST. Their results showed that the integrated treatments are significant since it could obtain more complete resections and better disease-free and overall survival [23]. Based on these reports, we could suggest lower rectal GIST patients with large volume and special location to attempt transvaginal resection combined with preoperative and postoperative imatinib treatment. These two patients failed to receive preoperative imatinib treatment although the tumors were resected with clear margin. The prognosis for these patients needs to be detected. Two patients should be followed up every 3 months. In summary, we consider the transvaginal approach for rectal GIST tumors as a reasonable surgical procedure.

## Conclusion

Transvaginal excision may be a minimally invasive and safe alternative for the management of rectal GIST in a lower location.

## Data Availability

The data and materials can be made available upon inquiry.

## Abbreviations

GIST: Gastrointestinal stromal tumor
IM: Imatinib mesylate
MR: Magnetic resonance
TEM: Transanal endoscopic microsurgery
